# Therapeutic potential and challenges of mesenchymal stem cells in neurological disorders: A concise analysis

**DOI:** 10.1093/jnen/nlaf021

**Published:** 2025-03-27

**Authors:** Enas H Bani Issa, Enas M Alghazo, Raghad Gharaibeh, Noor B Momani, Dana Z Taha, Renad J Jaradat, Ayman Alzu’bi, Fatimah A Almahasneh, Ejlal Abu-El-Rub, Raed M Al-Zoubi

**Affiliations:** Department of Basic Medical Sciences, Faculty of Medicine, Yarmouk University, Irbid, Jordan; Department of Basic Medical Sciences, Faculty of Medicine, Yarmouk University, Irbid, Jordan; Department of Basic Medical Sciences, Faculty of Medicine, Yarmouk University, Irbid, Jordan; Department of Basic Medical Sciences, Faculty of Medicine, Yarmouk University, Irbid, Jordan; Department of Basic Medical Sciences, Faculty of Medicine, Yarmouk University, Irbid, Jordan; Department of Basic Medical Sciences, Faculty of Medicine, Yarmouk University, Irbid, Jordan; Department of Basic Medical Sciences, Faculty of Medicine, Yarmouk University, Irbid, Jordan; Department of Basic Medical Sciences, Faculty of Medicine, Yarmouk University, Irbid, Jordan; Department of Basic Medical Sciences, Faculty of Medicine, Yarmouk University, Irbid, Jordan; Surgical Research Section, Department of Surgery, Hamad Medical Corporation, Doha, Qatar; Department of Biomedical Sciences, QU-Health, College of Health Sciences, Qatar University, Doha, Qatar; Department of Chemistry, Faculty of Science and Art, Jordan University of Science and Technology, Irbid, Jordan

**Keywords:** adipose tissue mesenchymal stem cells, Alzheimer disease, bone marrow mesenchymal stem cells, multiple sclerosis, Parkinson disease, stroke, umbilical cord mesenchymal stem cells

## Abstract

Neurological diseases comprise a wide array of conditions affecting both the central and peripheral nervous systems. Neurodegenerative diseases encompass a group of debilitating and often fatal neurological disorders for which effective treatments are currently lacking. Stem cells are recognized for their remarkable capacity for proliferation, multilineage differentiation, and self-renewal. The transplantation of stem cells represents a significant advancement in therapeutic strategies for neurological disorders, with applications in both preclinical and clinical settings. Mesenchymal stem cells (MSCs), in particular, have garnered substantial interest due to their unique properties, making them a highly sought-after source of therapeutic cells. Although the efficacy of MSCs in treating neurological disorders is well documented, further research is needed to elucidate the underlying mechanisms and to assess their in vivo applications more comprehensively. This article summarizes current research on the use of MSCs in the treatment of various neurological disorders, including Parkinson disease, stroke, multiple sclerosis, and Alzheimer disease.

## INTRODUCTION

Mesenchymal stem cells (MSCs) are multipotent stem cells capable of differentiating into various cell types. MSCs can be isolated from a variety of tissues, including umbilical cord, endometrial polyps, bone marrow, menstrual blood, and adipose tissue.^1^ They play a crucial role in the field of regenerative medicine, primarily due to their ability to support tissue regeneration, which depends on the secretion of trophic factors, and the ability to produce different mesenchymal lineages. Additionally, MSCs exhibit homing capabilities and possess trophic effects that can modulate the immune response, alter the microenvironment surrounding injured tissues, and promote tissue repair.^2^ In general, the therapeutic mechanisms of MSCs in neurological disorders are related to paracrine signaling (by both secretory activity and exosome [Exos] release), and they include immunomodulation, induction of endogenous neurogenesis, neuroprotection, transdifferentiation into neurons, angiogenesis, and extracellular matrix remodeling.^3^ Immunomodulation by MSCs involves both interaction with immune cells and the secretion of cytokines such as IL-6, hepatocyte growth factor, prostaglandin 2, and TGF-β1, which suppress immune response and inhibit neuron apoptosis.^4^ MSCs also contribute to synapse formation and neural growth through the expression of GAP43 protein, synapsin I, and NMDA receptors, among others.^5^ In addition, MSCs enhance injury repair through the release of numerous growth factors, cytokines, chemokines, and proteins, which exhibit proangiogenic, anti-apoptotic, and/or neuroprotective effects.^6^

Given these properties, MSCs hold promises for treating various neurological diseases. Neurological disorders represent the primary source of both physical and cognitive disabilities worldwide, currently impacting around 15% of the global population.^7^ Over the past 3 decades, there has been a significant increase in both the absolute number of fatalities and individuals experiencing disabilities due to neurological conditions, especially in low- and middle-income nations. Projections indicate that these figures will continue to rise worldwide, driven by factors such as population growth and an ageing demographic.^7^ Therefore, there is a pressing need for special and safe treatment options for these disorders. In this article, we review the use of MSCs from different sources in the treatment of Alzheimer disease (AD), Parkinson disease (PD), multiple sclerosis (MS), and stroke.

### MSCs and AD

Alzheimer disease is a chronic neurodegenerative disorder characterized by memory loss, cognitive dysfunction, and behavioral changes that hinder the patient's independence in daily activities. It is considered to be a multifactorial disease, involving a combination of genetic, molecular, and environmental factors that lead to progressive neuronal dysfunction and cell death in the brain.^8^

The core neuropathology of AD includes the accumulation of tau protein and β-amyloid. This accumulation results from a loss of regulation over the amyloid precursor protein (APP), a transmembrane protein normally cleaved by an α-secretase enzyme. The accumulation of amyloid-β in the extracellular space of neurons forms insoluble plaques that play a crucial role in neurotoxicity and impairment of neural function by disrupting cell-to-cell communication and initiating a neuroinflammatory response.^9^ Subsequently, hyperphosphorylated tau protein forms neurofibrillary tangles within neurons, interfering with their structural integrity and transport systems, ultimately leading to cellular dysfunction and death.^10^ These pathological processes are further compounded by neuroinflammation, oxidative stress, and injury to cholinergic neurons.^11^

Despite the seriousness of the disease, there is currently no drug that halts its progression or provides a cure. Traditional treatments focus mainly on alleviating symptoms and improving the quality of life for patients and caregivers. These drugs include cholinesterase inhibitors and N-methyl-D-aspartate (NMDA) antagonists.^8^ Various types of MSCs, including bone marrow-derived, adipose tissue-derived, and umbilical cord-derived MSCs, are being studied as potential therapies for AD due to their regenerative potential and ability to modulate immune responses. Key mechanisms of action include neuroprotective effects through the secretion of neurotrophic factors, anti-inflammatory properties that reduce neuroinflammation,^12^ promotion of neurogenesis,^13^^,^^14^ and the involvement of extracellular vesicles in cellular communication and brain repair, thereby improving cognitive performance.^15^

### ADSCs and AD

The efficacy of adipose-derived stem cells (ADSCs) in reducing the progression of AD has been demonstrated by many preclinical studies. Ma et al showed that intracerebral transplantation of ADSCs in amyloid β precursor protein/presenilin 1 (APP/PS1) double transgenic mice was effective in reducing the pathological aggregation of β-amyloid (Aβ). This reduction in amyloid plaques led to a decrease in the symptoms of AD, the restoration of memory function, and modulation of microglial function, ultimately slowing the progression of the disease.^16^ An experimental study conducted by Yan et al proposed that the transplantation of ADSCs reduced oxidative stress and cognitive impairment in mice. Additionally, it increased the number of BrdU(+)/Dcx(+) neuroblasts, indicating enhanced neurogenesis in APP/PS1 mice.^17^

The antioxidant properties of melatonin are widely recognized. Administering melatonin to ADSCs prior to transplantation has been found to be more effective in reducing Aβ plaques and microglial cell counts compared to ADSCs alone. This reduction positively impacts memory and cognition.^18^

ADSC-derived Exos have been investigated in the APP/PS1 mouse model of AD. Intranasal administration of these Exos has shown quick and efficient brain delivery, leading to a reduction in Aβ deposition, decreased microglial activation, and an increase in the number of newly born neurons. Furthermore, the Exos contain neuroprotective proteins such as neprilysin, neuroplastin, and eLFsA, which promote neurite outgrowth and neurogenesis.^19^ Neprilysin is an Aβ-degrading enzyme in the brain. Katsuda et al reported that delivering this protein via ADSC-derived Exos leads to a decrease in Aβ levels in N2a cells.^20^

Taken together, these findings suggest that ADSCs hold promise for AD therapy due to their ability to reduce Aβ plaques, modulate microglial function, promote neurogenesis, and deliver neuroprotective proteins.

### BMMSCs and AD

The efficacy of bone marrow-derived stem cells (BMMSCs) for managing the symptoms of AD has been reported in vivo. Qin et al demonstrated that the use BMMSCs transplantation in AD model mice can reduce neuropathological changes and improve cognitive deficits through various mechanisms, including inhibition of apoptosis and inflammation, neurogenesis, angiogenesis and immunomodulation. These mechanisms may have different effects at different stages of the disease. Specific genes have been identified as responsible for neuropathological characteristics in AD, which may aid in creating gene-specific patterns. Furthermore, the transplantation of BMMSCs could potentially alter gene expression.^21^ Li et al found that rat bone marrow stem cells were able to differentiate into neuron-like cells, partially expressing choline acetyltransferase (ChAT).^22^ Nestin, a marker of neural precursors, was also present in these neural cells, and there was an increase in brain nestin expression following BMMSCs treatment.^23^ In the hippocampus, various stages of neurogenesis were observed, including proliferation, differentiation, migration, targeting, and integration.^24^ It is worth mentioning here that although neurogenesis has been extensively studied and its mechanisms described, it is paramount to consider its complexity, the controversy around its occurrence and role in humans, and the differences between human and animal neurogenesis^25^ when addressing it as a mechanism of the MSCs effects. While the exact mechanism is still unclear, the transplanted stem cells may positively impact cell growth phases. MSCs have been found to produce trophic factors such as brain-derived neurotrophic factor (BDNF), nerve growth factor (NGF), and insulin-like growth factor 1 (IGF-1).^26^ These factors contribute to the recovery of neurobehavioral function and the stimulation of endogenous regeneration.

Yu et al tested the impact of BMMSC administration on seladin-1, a neuroprotective marker, and nestin expression in AD models. They determined that the transplantation of BMMSCs increased the levels of seladin-1 and nestin, possibly by activating the phosphoinositide 3-kinase/protein kinase B (PI3K/Akt) and extracellular signal-regulated kinases (ERK1/2) pathways. These studies provide initial support for the idea that utilizing BMMSCs could be a promising therapeutic strategy for combating neurodegeneration in conditions such as AD.^27^

### hUCB-MSCs and AD

Another important subtype of MSCs that may also be a potential treatment for AD is isolated from the umbilical cord. Jia et al performed a study using senescence accelerated mouse-prone 8 (SAMP8) mouse model which mimics accelerated aging in AD, in which human umbilical cord-derived mesenchymal stem cells (hUCB-MSCs) were utilized. The study revealed that the secretion of hepatocyte growth factor from hUCB-MSCs aids in the restoration of damaged nerve cells. This restoration was achieved through various mechanisms, such as downregulating hyperphosphorylated tau protein, reversing dendritic spine loss, promoting synaptic plasticity in the aged hippocampus, improving neurofibrillary tangles, and enhancing the cMet-AKT-GSK3 pathway, all of which collectively contributed to the restoration of cognitive function.^28^ Another study involved the administration of MSCs into the brains of AD mouse models. These cells produced a set of neurotrophic factors that could potentially aid in addressing the pathological processes of AD, rather than promoting the differentiation of neurons and glial cells.^29^ hUCB-MSCs have been proposed to release soluble intracellular adhesion molecule-1 (SICAM-1), which has a paracrine stimulatory effect on microglial cells, leading to the overexpression of neprilysin and a subsequent reduction in amyloid-β (Aβ) levels.^30^ Additionally, hUCB-MSCs secreted galectin-3, which offers protection against Aβ neurotoxicity and subsequently promotes the survival of neuronal cells.^31^ Furthermore, hUCB-MSCs boosted endogenous adult neurogenesis and synaptic activity through the secretion of growth differentiation factor 15 (GDF-15) and activin A.^32^

**Figure 1.**
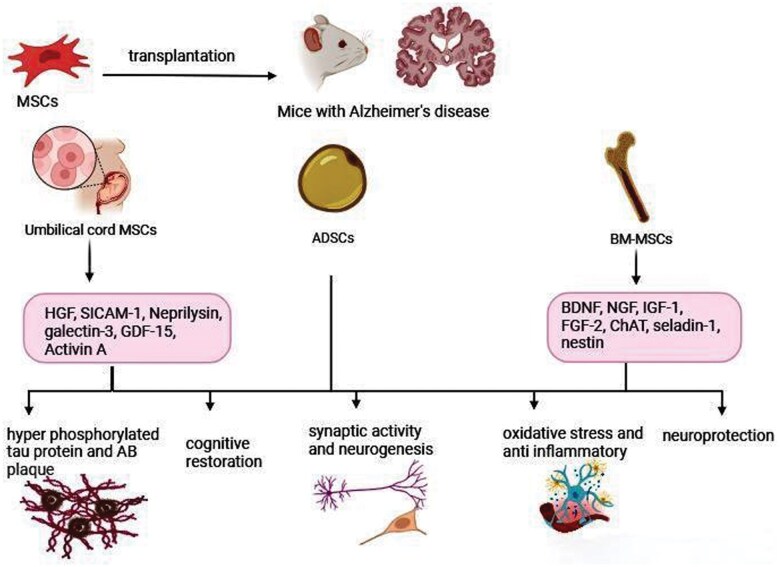
Suggested mechanisms of MSCs in halting or reversing the progression of Alzheimer disease. HGF: hepatocyte growth factor; SICAM-1: soluble intercellular adhesion molecule-1; GDF-15: growth differentiation factor; BDNF: brain-derived neurotrophic factor; NGF: nerve growth factor; IGF-1: insulin-like growth factor-1; FGF-2: fibroblast growth factor; ChAT: choline acetyltransferase.

By comparing different types of MSCs, there is currently no evidence to confirm which type may be the most effective in treating AD. However, Kern et al reported that there were no significant differences in morphology and immune response among the various types in general. The success rate of isolation was found to be higher for bone marrow and adipose tissue MSCs compared to umbilical cord blood MSCs. Additionally, colony frequency was highest in adipose tissue and lowest in hUCB-MSCs. Despite this, hUCB-MSCs can be cultured for a long time and exhibit the highest proliferation capacity, whereas BMMSCs showed the lowest.^33^ Notably, Naaldijk et al reported that BMMSCs could be more effective than ADSCs in treating AD.^34^ However, further research is needed to compare the therapeutic efficacy of different types of MSCs in treating AD. Figure 1 summarizes the potential mechanisms of MSCs in halting or reversing the progression of AD.

### MSCs and stroke

Stroke is a medical condition that impacts the nervous system, primarily as a consequence of the obstruction of blood vessels. The presence of clots in the brain interferes with normal blood circulation, leading to the blockage of arteries and potential ruptures, which can result in hemorrhage. When arteries in the brain rupture during a stroke, there is a sudden death of brain cells due hypoxia. Furthermore, stroke may also play a role in the onset of depression and dementia, and it is recognized as the second leading cause of death globally. The risk of experiencing a stroke increase with age; it doubles once individuals reach the age of 55. Ischemic occlusions are responsible for approximately 85% of stroke-related deaths, while the remaining cases are due to intracranial bleeding.^35^

Significant factors in the pathology of stroke include inflammation, energy depletion, disruption of homeostasis, acidosis, elevated intracellular calcium concentrations, excitotoxicity, toxicity mediated by free radicals, cytotoxicity driven by cytokines, activation of the complement system, dysfunction of the blood-brain barrier, stimulation of glial cells, oxidative stress, and the infiltration of leukocytes.^36^ The primary emphasis of current stroke treatments is on neuroprotection and reopening blocked blood vessels in the brain. This involves the use of hypothermia and the administration of antithrombotic, antiplatelet, and antihypertensive medications to address ischemic brain damage resulting from an embolism.^37^ Over the past 20 years, stem cells have shown promise in the treatment of stroke due to their ability to self-renew and develop into various types of cells.^38^ Research on stem cell therapy for ischemic stroke has primarily focused on 2 distinct mechanisms: (1) substituting damaged neural cells and tissues, and (2) paracrine functional effects, such as immunomodulation, pro-angiogenesis, and neuroprotective and neurotrophic functions.^38^ In general, the primary cell types that have undergone extensive preclinical and clinical testing in the context of regenerative therapy for ischemic stroke include embryonic stem cells (pluripotent stem cells), fetal stem cells (predominantly those derived from the fetal brain or spinal cord), adult stem cells (such as MSCs that are tissue-specific), and induced pluripotent stem cells (somatic cells reprogrammed to become pluripotent stem cells).^38^ Various cell replacement strategies have been suggested and evaluated in numerous stroke models throughout decades of research involving animal subjects. Furthermore, they have demonstrated advantageous paracrine effects, which may mitigate cell death and offer growth or trophic support to host cells, thereby enhancing regenerative processes within the host brain.^39^

### ADSCs and stroke

ADSCs have shown a significantly positive impact on neural function when utilized in a rodent stroke model. In a study by Kuang et al, adipose tissue was collected from wild-type C57BL/6 mice; the data indicated that ADSCs‐extracellular vesicles of native origin offered neuroprotection and improved neurological rehabilitation through the suppression of autophagy induced by ischemia. This suppression of autophagy is facilitated by extracellular vesicles transferring miR‐25‐3p from ADSCs to their target cells.^40^

In other studies, it has been shown that the administration of ADSCs has the potential to stimulate the generation of nerve cells and activate brain repair markers linked to neurogenesis, aiding in recovery from chronic stroke.^41^ In stroke treatment, ADSCs release vascular endothelial growth factor (VEGF) to initiate the angiogenesis process, which may serve as a protective mechanism for neurons by preventing cell death.^42^ Additionally, the utilization of ADSCs in stroke therapy reduces the generation of glial fibrillary acidic protein-positive cells, ultimately preventing astrogliosis in the brain.^43^^,^^44^ Another potential mechanism for the protective role of ADSCs may involve an immunomodulatory process leading to increased interleukin-10 and decreased tumor necrosis factor-α levels in rats with middle cerebral artery occlusion.^45^ However, concerns regarding the risk of tumor formation and the low survival rate of transplanted cells limit the use of ADSCs.^46^

### BMMSCs and stroke

BMMSCs have been studied for their potential in stroke treatments following the revelation that they can differentiate into neural and glial cells in vitro.^47^ Subsequent in vivo experiments revealed that BMMSCs, when transplanted intracerebrally into rat stroke models, were able to migrate to the ischemic brain damage locations and transform into neural cells, resulting in enhanced recovery.^48^ Initially explored for their ability to generate new neurons, further research has revealed that BMMSCs do not provide therapeutic advantages through neuronal substitution. Instead, BMMSCs have been found to release substances that support the growth of new nerve cells and reduce inflammation, thereby improving natural recovery processes.^49^

More than 12 clinical trials utilizing BMMSCs for stroke treatment are currently in progress or have been finalized. The majority of these clinical trials focus on BMMSCs administered via intra-arterial (IA) or intravenous (IV) routes, which may indicate their enhanced safety profile and comparatively less demanding technical prerequisites relative to intracerebral transplantation procedures.^49^ BMMSCs contribute to the management of strokes through various mechanisms, such as facilitating cell migration, promoting angiogenesis,^50^ inhibiting apoptosis,^51^ secreting neurotrophic factors,^52^^,^^53^ reconstructing neural circuits,^54^ and modulating immune responses.^55^ The transplantation of BMMSCs improves the formation of new blood vessels in areas affected by inadequate blood supply, leading to an increase in the number of small blood vessels and improving neurovascular injuries.^56^

BMMSCs are capable of releasing VEGF, basic fibroblast growth factor (bFGF), and placental growth factor, as documented by multiple studies.^53^^,^^57^ Mitochondrial transport via tunneling nanotubes could be a crucial mechanism employed by BMMSCs to safeguard mitochondrial function and enhance angiogenesis.^58^ Another study reported that BMMSCs may facilitate connections between surrounding cells, such as astrocytes and endothelial cells.^59^ Also, it has been found that BMMSCs could uphold the blood-brain barrier's integrity, create a supportive microenvironment for neurogenesis, and enhance recovery of neurological function.^59^ Bioactive compounds originating from BMMSCs promote neurogenesis, enhance white matter integrity, and stimulate synaptogenesis.^60^

BMMSCs transplantation did not result in negative effects, including venous thromboembolism, abnormal cell proliferation, systemic cancer, systemic infection, or neurological decline,^61^ thus endorsing the utilization of these cells in therapy. However, the full elucidation of these mechanisms remains incomplete. Further investigation is necessary to ascertain the molecular biological processes involved in neural plasticity and angiogenesis.

### hUCB-MSCs and stroke

The transplantation of MSCs derived from the human umbilical cord (hUCB-MSCs) represents a promising treatment option for acute ischemic stroke (AIS).^62^ hUCB-MSCs have the ability to prevent the entry of inflammatory cells into the brain tissue and enhance the restoration of brain tissue integrity and functionality. Administering a high dosage of hUCB-MSCs early on can significantly improve the restoration of neurological and motor function and decrease the extent of the stroke.^63^ In the course of ischemic stroke treatment, hUCB-MSCs are directed to the site of brain injury caused by hypoxia through a homing mechanism facilitated by stromal cell-derived factors and chemokine receptors.^64^ These MSCs perform their roles by engaging in immunomodulation, reducing inflammation, preventing apoptosis, promoting vascular regeneration, and aiding in neural repair and remodeling.^60^^,^^63^

In recent studies, it was found that the administration of hUCB-MSCs resulted in a notable decrease in concentrations of the pro-inflammatory interleukins (IL-1β, IL-6) and tumor necrosis factor-α (TNF-α) while simultaneously increasing the serum levels of the anti-inflammatory factor IL-10 in the serum.^64^ Based on these findings, the administration of hUCB-MSCs demonstrated effective immunomodulatory capabilities in treating stroke, enhancing the production of chemokines and neurotrophic factors during the sub-acute phase. Additionally, hUCB-MSCs administration increased the levels of chemokines, suggesting that hUCB-MSCs encourage the restoration, growth, and development of neurons.^64^

Furthermore, hUCB-MSCs, when influenced by the SDF-1α/CXCR4 axis, can travel to the blood-brain barrier. Once there, they release neurotrophic factors via paracrine signaling, which helps prevent nerve cell apoptosis in brain tissue and aids in the recovery of brain structure and function.^65^ Although hUCB-MSCs offer significant benefits when compared to other types of MSCs,^62^ their limited therapeutic impact during later time frames may be due to the liquefaction of injured tissues and the development of cysts that are not easily eliminated by the body in the subacute and sequelae phases of stroke. Additionally, the inflammatory responses and the presence of glial scars during this period might hinder the functionality of stem cells,^64^ and the specific mechanisms of the treatment are not yet fully understood.

**Figure 2.**
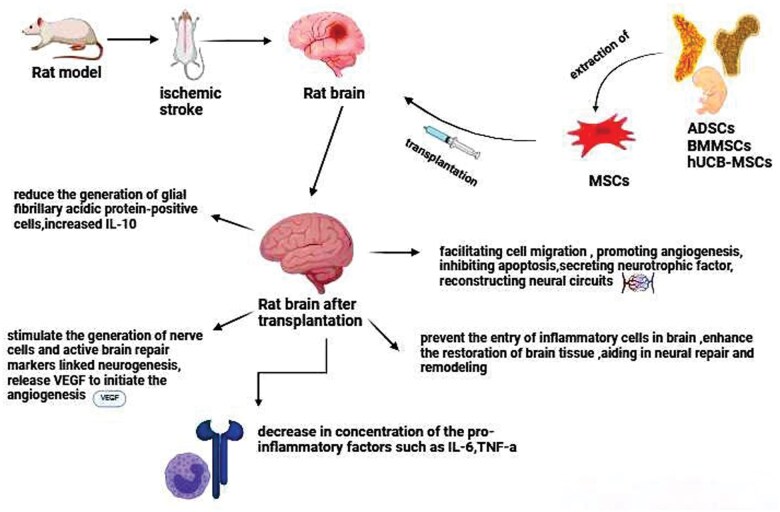
Suggested mechanisms of different types of MSCs that have been studied for the treatment of stroke. MSCs: mesenchymal stem cells; ADSCs: adipose stem cells; BMMSCs: bone marrow mesenchymal stem cells; hUCB-MSCs: human umbilical mesenchymal stem cells; IL-6: interleukin-6; TNF-α: tumor necrosis factor-α; VEGF: vascular endothelial growth factor; IL-10: interleukin-10.

The use of BMMSCs in stroke treatment has been found to be safe, yet not effective in clinical trials. MSCs are a diverse group, and newly identified types like hUCB-MSCs may have the potential to be more effective.^66^ Despite the need for further exploration, stem cell therapies for stroke show potential in safeguarding and promoting the regeneration of neurons, thus enhancing the prognosis for stroke survivors.^49^  Figure 2 highlights the suggested mechanisms of different types of MSCs in the treatment of stroke.

### MSCs and MS

MS is an autoimmune disorder that persistently affects the central nervous system, leading to inflammation, degradation of myelin, and injury to nerve cells.^67^ Oligodendrocytes, the cells responsible for the production and maintenance of myelin, come under attack from the immune system, resulting in thinning and eventual loss of the myelin sheath. The degradation of myelin progressively causes electrical instability, disrupts axonal conduction, and leads to functional abnormalities throughout the neuron. This sequence of events begins with inflammation and culminates in axonal damage and dysfunction.^68^

Hormones and immunosuppressants are frequently employed in the management of MS. Nevertheless, prolonged administration of these substances can lead to adverse effects and incur significant costs. Additionally, patients often experience a resurgence or exacerbation of symptoms upon discontinuation of medication.^69^ Modern therapeutic approaches primarily focus on alleviating symptoms and managing the progression of the disease. However, the necessity to improve rehabilitation and neurological recovery methods propels the quest for more effective treatment options.^70^

In recent times, stem cell therapy has emerged as a promising avenue for managing MS. Stem cells, known for their unique ability to self-renew and differentiate into various cell types, offer significant potential for the regeneration of injured neural tissue, modulation of immune responses, and enhancement of conditions conducive to natural repair processes.^71^

### ADSCs and MS

A research study carried out by Ragerdi et al investigated the impact of administering ADSCs combined with 17β-estradiol in a cuprizone mouse model of demyelination and remyelination, which has been used to study specific aspects of MS pathology.^72^ The findings indicated that this injection effectively stimulated myelin production. While the precise mechanisms underlying these therapeutic effects remain unclear, 2 primary hypotheses have been proposed: first, that ADSCs, similar to MSCs, may differentiate into mature oligodendrocytes capable of producing myelin and second, that they may enhance the survival and proliferation of endogenous precursor cells through indirect pathways, potentially by activating restorative and regenerative processes in the brain via interactions with MSCs.^73^

Additionally, Constantin et al proposed that MSCs have the ability to secrete several growth factors, including bFGF, brain-derived neurotrophic factor (BDNF), and platelet-derived growth factor (PDGF), all of which significantly facilitate the process of oligodendroglial differentiation.^74^

### BMMSCs and MS

Many preclinical studies highlighted the therapeutic efficacy of BMMSCs in animal models of MS. Abi Chahine et al studied the therapeutic effects of infusing BMMSCs in alleviating MS; their study indicated that BMMSCs injections led to significant improvements in MS patients with minimal side effects.^75^

### hUCB-MSCs and MS

In a study by Liu et al, myelin oligodendrocyte glycoprotein (MOG-34-56) was administered to monkeys to induce experimental autoimmune encephalomyelitis (EAE), a model of MS.^76^ The monkeys were then treated with hUCB-MSCs, which were found to effectively prevent clinical symptoms and sustained their therapeutic effects throughout the treatment period. Histological examination of brain tissue sections demonstrated the presence of a healthy myelin layer and the absence of inflammatory processes. Furthermore, these cells promoted the expression of immune regulatory cytokines, including IL-17, IL-13, IL-10, IFN-γ, and VEGF, while simultaneously reducing pro-inflammatory cytokines such as SCD40L and IL-5.^76^

Bone marrow-derived mesenchymal stem cells (BMMSCs) are the most extensively researched MSCs and have the potential to enhance outcomes in both remitting chronic and relapsing EAE.^77^ Despite the promising results associated with BMMSCs, their multipotent characteristics may pose limitations to their practical use. In contrast, hUCB-MSCs have emerged as a more suitable alternative for the treatment of MS due to their ability to undergo differentiation, modulate immune responses, and promote tissue regeneration.^78^ Research indicates a reduction in the quantity of inflammatory cells and infiltrating astrocytes following injury; however, the application of hUCB-MSCs therapy may be restricted.^76^

**Figure 3.**
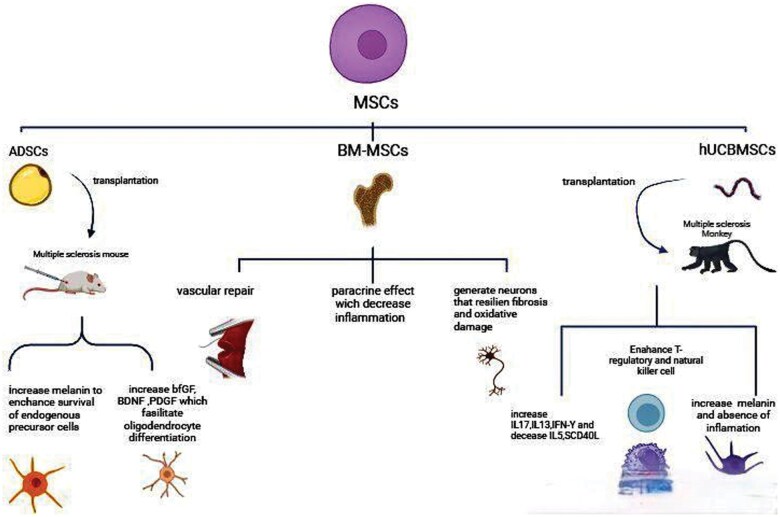
Impact of various types of mesenchymal stem cells (MSCs) in the treatment of multiple sclerosis through multiple mechanisms. MSCs: mesenchymal stem adipose-derived stem cells; BM-MNSCs: bone marrow-derived mesenchymal stem cells; hUCBMSCs: human umbilical cord blood-derived mesenchymal stem cells; BDNF: brain-derived neurotrophic factor; PDGF: platelet-derived growth factor; IL: interleukin; IFN-Y: interferon gamma.

Additionally, adipose tissue has emerged as a valuable source of cells for medical applications, being rich in ADSCs, which are readily obtainable in substantial quantities and easily accessible.^79^ ADSCs demonstrate many of the same beneficial properties as BMMSCs, including differentiation capacity, inhibition of T-cell activation and proliferation, production of anti-inflammatory factors, and facilitation of tissue repair through cytokine secretion that may be relevant to MS treatment.^80^  Figure 3 visualizes the impact of various types of MSCs in the treatment of MS and the suggested mechanisms.

### MSCs and PD

Parkinson disease is a neurodegenerative disorder characterized by the degeneration of dopamine-producing neurons and a reduction in dopamine levels across several neural networks. This neuronal loss is associated with the buildup of Lewy bodies, which are clumps of α-synuclein protein that disrupt the normal functioning of these neurons. The most affected network is the nigrostriatal pathway, specifically involving the substantia nigra pars compacta and the striatum.^81^ PD is linked to several risk factors, including advancing age, genetic factors, pesticide exposure, and environmental toxins like synthetic heroin. However, the precise causes of the disease are not fully understood.^82^

Parkinson disease is characterized by a range of motor and non-motor symptoms. Motor symptoms typically include resting tremors, rigidity, bradykinesia (slowness of movement), and stooped posture. Non-motor symptoms may involve neurobehavioral disorders such as depression and anxiety, cognitive impairment including dementia, and autonomic dysfunctions such as orthostatic hypotension and hyperhidrosis.^82^

There is no definitive treatment for PD; all current treatments focus on delaying the progression of the disease, alleviating symptoms, and improving quality of life.^83^ In addition, traditional treatments for PD have several severe side effects, which necessitate the exploration of alternative treatment methods, such as the use of MSCs.

Researchers have been studying the clinical potential of MSCs derived from adult bone marrow, umbilical cord, adipose tissue, and other sources for tissue repair and treatment of a wide range of diseases due to their ability to differentiate, expand in vitro, release trophic factors, and exhibit immunoregulatory properties.^84^

### ADSCs and PD

Adipose-derived stem cells (ADSCs) exhibit a surface antigen marker profile and differentiation capacity that closely resemble those of BMMSCs, while also displaying greater heterogeneity.^85^ They also possess a wide-ranging immune regulatory capacity, rendering them excellent candidates for cell-based therapy.^86^

A piece of evidence suggests that MSCs can migrate to sites of inflammation through chemotaxis. They exert an immunomodulatory effect by influencing specific chemotactic recruitment responses, which helps decrease inflammation in the damaged area and supports tissue repair.^87^^,^^88^

In a study performed by Schwerk et al, the immediate neuroprotective and neurogenic effects of ADSCs were evaluated using the 6-hydroxydopamine (6-OHDA) model of PD in rats. The study found a significant increase in subventricular neurogenesis in MSC-transplanted rats compared to controls, with most MSCs localized in the substantia nigra and adjacent arachnoid mater.^89^ PD patients often experience reduced dopaminergic activity in the subventricular zone (SVZ), which disrupts the SVZ-olfactory bulb (OB) axis and contributes to hyposmia. Transplanting MSCs may offer a way to directly modulate this axis.^89^ ADSC-induced effects encompassed enhanced memory performance, elevation of peripheral anti-inflammatory cytokines, maintenance of dopamine levels, and promotion of neurogenesis in the hippocampal and SVZ areas. Nevertheless, the improvement in cognitive abilities did not correspond to enhanced motor functions.^90^

### BMMSCs and PD

Many studies highlight the beneficial effects of BMMSCs in PD models. Bouchez et al performed an intracerebral injection of 6-OHDA in rats to induce PD symptoms, followed by injection with rat BMMSCs. After transplantation, the count of rotations induced by amphetamine, caused by the amphetamine-induced positive regulation of dopamine levels in the synapse, showed a notable decrease, while the presence of dopaminergic markers in nerve terminals and cell bodies was partially replenished. Additionally, the levels of dopaminergic biomarkers in nerve endings and cell bodies experienced a substantial recovery. This outcome was linked to the preservation of dopaminergic neurons and the emergence of new growth from the remaining nigrostriatal fibers.^91^

BMMSCs have the ability to migrate to the injured brain, leading to a notable decrease in serum TGF-β1 levels, along with an increase in nestin gene expression and brain tyrosine hydroxylase levels. Additionally, levels of dopamine (DA) in the brain and serum BDNF were elevated. The anti-inflammatory, immunomodulatory, and neurotrophic effects of BMMSCs are believed to play a role in restoring these markers to normal levels.^92^

The recognized ability of BMMSCs to release neurotrophic mediators, such as BDNF, NGF, neurturin, and glial-derived neurotrophic factor (GDNF), is crucial for neurogenesis, neuroprotection, neuronal survival, and differentiation.^93^ Furthermore, BMMSCs can transform into dopaminergic precursors, providing similar characteristics, thereby enhancing behavior in parkinsonian rats following transplantation.^94^

### hUCB-MSCs and PD

Several studies have shown that hUCB-MSCs provide neuroprotection, but their use is often hindered by issues such as uncontrolled differentiation. Stem cells communicate with other cells via secreted Exos. In addition, hUCB-MSCs have been successfully isolated, characterized, and shown to promote the proliferation of 6-OHDA-stimulated subline of human neuroblastoma (SK-N-SH; SH-SY5Y) cells.^95^ hUCB-MSCs secrete growth factors, cytokines, and Exos that play crucial roles in neuroprotection, neurodifferentiation, apoptosis reduction, and inflammation regulation. Importantly, the size of Exos enables them to cross the blood-brain barrier, thereby enhancing their therapeutic potential for treating CNS disorders.^95^

In a trial by Huang et al, hUCB-MSCs were cultured in vitro, and then hUCB-MSC-derived Exos were obtained. A mouse model of PD exhibiting both motor and non-motor symptoms, such as olfactory impairment, was tested in various ways to examine any changes in motor and non-motor functions while administering hUCB-MSC-Exos via a nasogastric route.^96^ Over time, intranasal administration of hUCB-MSC-Exos enhanced both motor and non-motor functions in the mice.^96^ Nevertheless, a challenge remains, as only a small fraction of transplanted hUCB-MSCs has been observed in the target tissue and these cells exhibit low survival rates in the host.^97^

**Figure 4.**
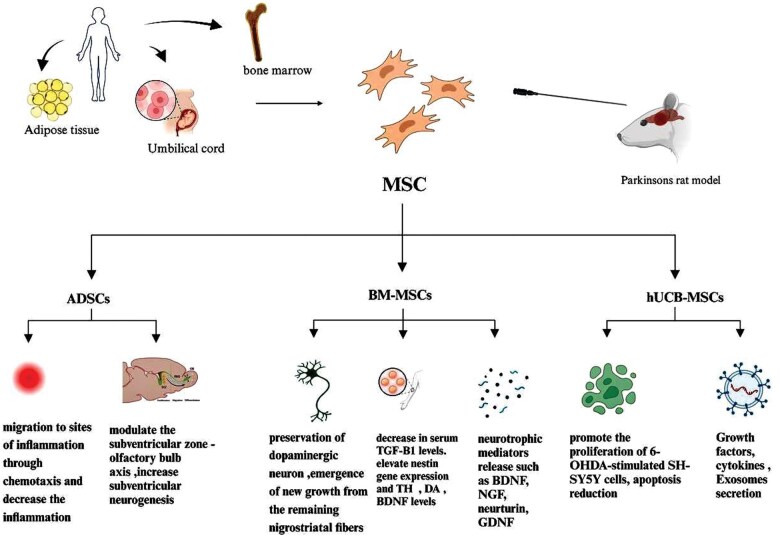
A visual overview of the therapeutic potential and proposed mechanisms of different types of mesenchymal stem cells in Parkinson disease. MSCs: mesenchymal stem cells; BMMSCs: bone marrow mesenchymal stem cells; hUCB-MSCs: human umbilical mesenchymal stem cells; ADSCs: adipose-derived stem cells; TH: tyrosine hydroxylase; DA: dopamine; BDNF: brain-derived neurotrophic factor; NGF: nerve growth factor; GDNF: glial-derived neurotrophic factor; SH-SY5S: subclone human-SY5Y; 6-OHDA: 6-hydroxydopamine.

While various types of MSCs show promise in treating PD, each type has unique advantages and limitations. BMMSCs have frequently been used in clinical trials,^98^ but hUCB-MSCs offer faster self-renewal capabilities and a painless collection procedure.^99^ Overall, continued research and clinical trials are essential to determine the most effective MSC type for PD treatment, optimize therapeutic approaches, and ensure long-term efficacy and safety. Figure 4 summarizes the therapeutic potential and proposed mechanisms of different types of MSCs in PD.

## CONCLUSION

Stem cell therapy has shown promising results in treating neurological conditions such as AD, stroke, MS, and PD. A comparison of various MSCs reveals a lack of conclusive evidence regarding which type is most effective for treating neurological disorders.

BMMSCs may demonstrate greater efficacy than ADSCs in treating AD. While different MSCs types show comparable potential in treating PD, hUCB-MSCs are noted for their rapid proliferation and the ease of their collection process. Also, hUCB-MSCs may possess greater therapeutic potential in treating stroke. ADSCs have proven to be an effective type of MSC in an MS model. Despite their demonstrated effectiveness, several challenges and barriers hinder the use of these stem cells for these neurological conditions, such as the risk of tumor formation, immune rejection, and potential toxicity. Furthermore, the ideal route of transplantation and the necessary procedures for optimization have not been determined, highlighting the need for further research to thoroughly investigate the interactions between the transplantation process and surrounding tissues.

## Data Availability

The data that support the findings in this study are available from the corresponding author upon reasonable request.
